# Expression of XPG Protein in the Development, Progression and Prognosis of Gastric Cancer

**DOI:** 10.1371/journal.pone.0108704

**Published:** 2014-09-30

**Authors:** Na Deng, Jing-wei Liu, Li-ping Sun, Qian Xu, Zhi-Peng Duan, Nan-Nan Dong, Yuan Yuan

**Affiliations:** 1 Tumor Etiology and Screening Department of Cancer Institute and General Surgery, the First Affiliated Hospital of China Medical University, and Key Laboratory of Cancer Etiology and Prevention (China Medical University), Liaoning Provincial Education Department, Shenyang, China; 2 Department of Oncology, The Fourth Affiliated Hospital of China Medical University, Liaoning, Shenyang, China; University of Texas MD Anderson Cancer Center, United States of America

## Abstract

**Background:**

Xeroderma pigmentosum group G (XPG) plays a critical role in preventing cells from oxidative DNA damage. This study aimed to investigate XPG protein expression in different gastric tissues and in patients with diverse prognoses, thus providing insights into its role in the development, progression and prognosis of gastric cancer (GC).

**Methods:**

A total of 176 GC, 131 adjacent non-tumour tissues, 53 atrophic gastritis (AG) and 49 superficial gastritis (SG) samples were included. Immunohistochemical staining was used to detect XPG protein expression.

**Results:**

XPG expression was significantly higher in GC tissues compared with adjacent non-tumour tissues. In the progressive disease sequence SG→AG→GC, XPG expression was significantly higher in AG and GC compared with SG. Analysis of clinicopathological parameters and survival in GC patients demonstrated a significant association between XPG expression level and depth of tumour invasion, macroscopic type, Lauren’s classification, smoking, *Helicobacter pylori* infection and family history. Cox multivariate survival analysis indicated that patients with positive XPG expression had significantly longer overall survival (P = 0.020, HR = 0.394, 95%CI 0.179–0.866), especially in aged younger than 60 years (P = 0.027, HR = 0.361, 95%CI 0.147–0.888) and male patients (P = 0.002, HR = 0.209, 95%CI 0.077–0.571).

**Conclusions:**

This study demonstrated that XPG protein expression was related to the development, progression and prognosis of GC, and might thus serve as a potential biomarker for its diagnosis and prognosis.

## Introduction

Gastric cancer (GC) is the world’s fourth most common cancer and the second main cause of cancer-related death [1]. Despite recent advances in the diagnosis and therapy of GC, its incidence and associated mortality remain relatively high [2]. The risk factors for GC include genetic predisposition, *Helicobacter pylori* infection, and diet and lifestyle factors, etc, which can effect the development, progression and prognosis of GC.

Cellular DNA is constantly at risk of damage by endogenous and exogenous stimuli, leading to a dynamic balance between damage and repair. An imbalance between DNA damage and repair contributes to the initiation of cancer [3]. Oxidative DNA damage may lead to defects in transcription, and to duplication, mutation and genomic instability, which may in turn lead to cell dysfunction [4]. DNA-repair ability thus plays an essential role in maintaining the physiological functions of normal cells. The DNA-repair system consists of nucleotide excision repair (NER), base excision repair and mistmach repair. NER monitors and repairs a variety of DNA damages, such as ultraviolet-induced cyclobutane pyrimidine dimers, bulky adducts and DNA cross-links [5], [6], [7]. The process involves various enzymes including excision repair cross-complementing group (ERCC)1, XPD (ERCC2), XPF (ERCC4), XPG (ERCC5), XPC and ERCC6 (Cockayne syndrome B protein) [8]. It has been suggested that genomic instability is involved in tumour initiation, and multistep mutations occur throughout life [9]. NER is a versatile system able to repair multiple DNA damages caused by genetic instability, and thus plays an important role in the early formation of tumours.

Xeroderma pigmentosum group G (XPG) is a structure-specific nuclease belonging to the Fen1 family, which is encoded by *ERCC5* (excision repair cross-complementing group 5) [10], [11], [12]. XPG is an indispensable member of the NER pathway responsible for the 3′ excision of DNA damage in mammals [13]. Recent investigations have focused on the association between XPG and chemotherapeutic sensitivity. However, few studies have detected the expression of XPG protein in normal tissues and tumours. Although previous studies have been performed in the peripheral blood or metastatic cell lines, without considering expression profiles in paired tissues. In addition, no study to date has investigated the expression of XPG in cancer by immunohistochemical staining, especially in GC, atrophic gastritis (AG) and superficial gastritis (SG), and the association between XPG expression and the biological behaviour and prognosis of GC remains largely unknown.

In the present study, we detected XPG protein expression levels in tissues from patients with different gastric diseases by immunohistochemical staining, and explored its expression profiles in the disease sequence SG→AG→GC. We also investigated the relationships between XPG protein expression and clinicopathological parameters and survival in GC patients, to shed light on the potential roles of XPG in the development, progression and prognosis of GC.

## Materials and Methods

### Patients and tissue specimens

A total of 278 patients were enrolled from the Department of Surgical Oncology of the First Affiliated Hospital of China Medical University and from individuals who participated in a health-check program involving gastroscopy for GC screening in hospitals located in Zhuanghe and Shenyang in Liaoning Province, China, between 2008 and 2011. Tissue samples were obtained from 176 patients with histologically confirmed GC (including coupled adjacent non-tumour tissues from 131 cases), 49 patients with SG, and 53 patients with AG. Patients who (i) had synchronous or metachronous malignant tumours, (ii) XP disease, or (iii) underwent preoperative radiotherapy or chemotherapy were excluded from this study. Follow-up was completed by August 2013. All patients underwent endoscopic gastric mucosal biopsy. Biopsy specimens were paraffin embedded and stained with haematoxylin and eosin for histological diagnosis, which was accomplished by two experienced pathologists. There were no significant differences among the GC, SG, AG and adjacent non-tumour groups in terms of gender or age composition (*P* = 0.330 and *P* = 0.431, respectively) (Table 1). Patients were surgically staged according to the current Borrmann classification system. Histological results was determined on the basis of the World Health Organization criteria, and tumours were staged using the 7th edition of the TNM staging system of the International Union Against Cancer (UICC)/American Joint Committee on Cancer (AJCC) (2010), based on postoperative pathologic examination. A total of 176 patients were histologically confirmed with gastric adenocarcinoma; most cases could be classified according to Lauren classification, but 17 could not. Among the 176 GC cases, 63 were intestinal type, 96 were diffuse type and 17 were mixed type. History of drinking was defined as an average alcohol daily intake ≥50 g and continued ≥1 year. The end of the follow-up time is August 2013. In 176 cases patients, 169 cases completed follow-up information, and follow-up time ranged from 22 month to 38 months. 41 of the 169 patients (24.3%) with gastric cancer had died and the median overall survival time of all patients was 29 months. This study was approved by the Institute Research Medical Ethics Committee of the First Affiliated Hospital of China Medical University. Written informed consents were obtained from participants. Medical histories (including age, sex, smoking, and alcohol consumption) were obtained by questionnaire and the records were computerized.

**Table 1:** Clinicopathological parameters in adjacent, AG, SG, GC and survival in GC.

Variable	Categories				P	Cases of Events	MST	P
	Adjacent(131)	AG (53)	SG (49)	GC (176)				
Gender								0.837
Male	90	31	28	118	0.330	31	37	
Female	41	22	21	58		16	31.308*	
Age								0.548
<60	75	29	29	100	0.431	28	31.374*	
≥60	56	24	20	76		19	37	
Smoking								0.457
Yes	53	18	18	74	0.720	18	33.202*	
No	78	35	31	102		29	37	
Drinking								0.297
Yes	39	12	9	52	0.335	17	37	
No	92	41	40	124		30	32.823*	
HP infection status								0.817
positive	21	35	13	7	**0.001**	2	37	
negetive	5	18	36	30		8	33.429*	
Macroscopic Type								**<0.001**
Early stage				29		1	36.619*	
Borrmann I–II				23		3	34.25*	
Borrmann III–IV				115		43	37	
Lauren’s classification								0.154
intestinal-type				63		14	37	
Diffuse-type				96		31	37	
TNM stage								**<0.001**
I–II				80		9	35.380*	
III				87		38	36	
Lymph node metastasis								
Positive				105		39	37	
Negative				61		8	35.053*	**<0.001**
T stage								**<0.001**
T1				27		1	36.579*	
T2				27		3	34.630*	
T3				23		5	31.145*	
T4				90		38	36	
Growth pattern								0.201
Expanding				23		3	37	
Intermediate				78		23	36	
Infiltrative				66		21	30.216*	
Lymphatic invasion								0.184
Negative				33		12	36	
Positive				134		33	32.076*	
family history								0.204
Positive				32		6	32.924*	
Negative				135		41	37	

MST median survival time.

*mean survival time.

### Immunohistochemistry

Formalin-fixed, paraffin-embedded tissues were cut into 4-µm-thick sections and mounted on poly-L-lysine-coated glass slides. Briefly, slides were deparaffinized in xylene, rehydrated in a graded alcohol series and washed in tap water. The tissue sections were incubated in boiling sodium citrate buffer (pH 6.0) for 100 s in a steam pressure cooker for antigen retrieval. Endogenous peroxidase was blocked using 3% hydrogen peroxide for 10 min, and the sections were then washed with phosphate-buffered saline (PBS), pH 7.4. Tissue collagen was blocked to avoid nonspecific binding by the addition of 10% normal goat serum at 37°C for 10 min. The polyclonal antibody anti-XPG (ab-99248, 1∶300 dilution; Abcam, Cambridge, UK) was used as the primary antibody to detect XPG protein expression, and incubated for 4°C overnight. After rinsing three times with PBS for 5 min each, the sections were incubated with biotinylated secondary antibody (goat anti-rabbit antibody, Maixin Inc., Fujian, China) and streptavidin-biotin peroxidase for 10 min each at 37°C. The slides were then washed in PBS and stained with 3, 3-diaminobenzidine tetrahydrochloride and counterstained with haematoxylin. Finally, the sections were dehydrated and mounted. Primary antibodies were replaced with PBS buffer as a negative control.

### Evaluation of immunohistochemistry

The immunohistochemical results were evaluated and scored independently by two investigators who were blinded to the patients’ clinicopathological characteristics. Nuclear positivity for XPG protein was evaluated using a semi-quantitative scoring criterion based on the staining intensity (0, no staining; 1, light brown staining; 2, brown staining; and 3, heavy brown staining) and proportion of stained epithelial cells (0, ≤5%; 1, 5–25%; 2, 25–50%; 3, 50–75%; and 4, ≥75%). Staining intensity was measured at the sites of the antrum of the stomach and gastric body gland. The percentage positivity of epithelial cells and staining intensity were then multiplied to generate an immunoreactivity score (IS) for each specimen [14]. The expression was graded as: negative(–), score = 0; weak expression(+), score = 1–4; moderate expression(++), score = 5–8; and strong expression(+++), score = 9–12.

### Statistical analysis

Statistical analysis was performed using SPSS (16.0) statistical software (SPSS, Chicago, IL, USA). Non-parametric tests were used to analyse the differences in XPG expression in the SG-AG-GC sequence, and differences between GC and adjacent non-tumour tissues. Correlations between clinicopathological factors and XPG expression were analysed by the χ^2^ test or the Fisher’s exact probability test. Survival analysis was performed using Kaplan–Meier curves, and differences between the groups were analysed using the log-rank test. Cox regression analysis was conducted for multivariate analysis. Two-tailed *P* values<0.05 were considered statistically significant.

## Results

### Expression of XPG protein in gastric cancer and non-tumour tissues

**Figure 1:**
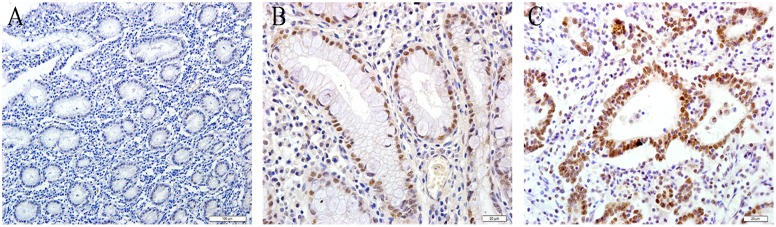
Representative photomicrographs of immunohistochemical staining of XPG in different gastric specimens. Low nuclear expression of XPG was observed in the antrum of the stomach in SG (a). XPG expression levels in AG (b) and GC (c) were higher than in SG. Original magnification, ×400.

**Figure 2:**
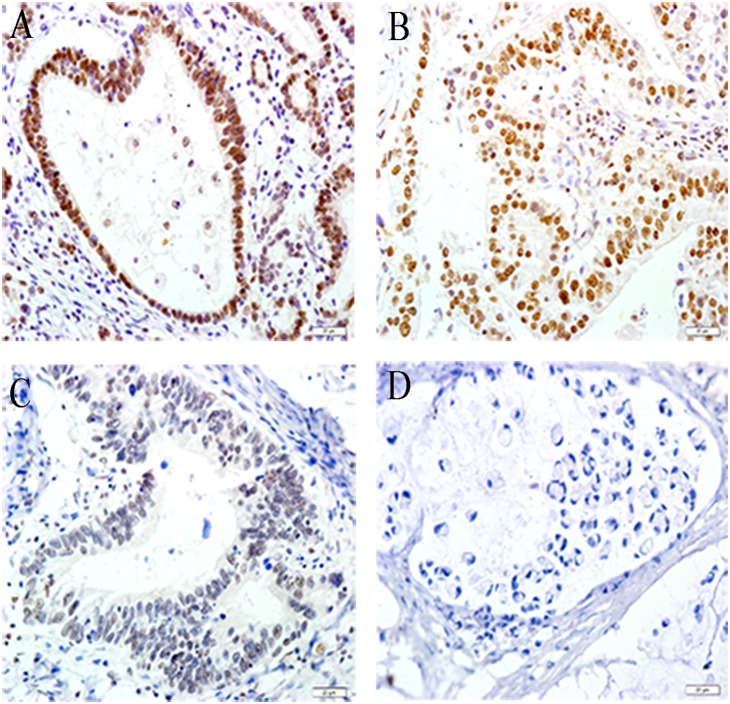
XPG expression in GC tissues. XPG staining in the nucleus was strongly positive (+++) in (a), moderately positive (++) in (b), weakly positive (+) in (c) and negative (−) in (d). Magnification, ×400.

XPG immunostaining demonstrated a predominantly nuclear localization (Figures 1 and 2). In the progression of gastric diseases, there were significant differences in XPG expression levels between AG and SG (*P1*<0.001), and between GC and SG (*P2* = 0.031). XPG expression was significantly higher in AG and GC than in SG, respectively (Mann–Whitney U-test test, Table 2). In addition, we found the expression levels of XPG in GC were significantly higher than in adjacent non-tumour tissues (P<0.001). At the same time, we classified adjacent non-tumour tissues into 41 cases AG and 88 cases SG. The results suggested that XPG expression was significantly higher in GC than its adjacent SG tissues (P<0.001); no significant association was observed between GC and its adjacent AG tissues (P = 0.244). The relationship of XPG expression in the samples of adjacent tissue and coupled GC were displayed in Table 3.

**Table 2:** XPG expression in SG, AG, GC.

		(–)	(+)	(++)	(+++)	PR (%)	
Group	Cases	n (%)	n (%)	n (%)	n (%)		
SG	49	9(18.4)	27(55.1)	9(18.4)	4(8.2)	81.6	ref.
AG	53	1(1.9)	17(32.1)	15(28.3)	20(37.7)	98.1	**P1<0.001**
GC	176	26(14.8)	69(39.2)	53(30.1)	28(15.9)	85.2	**P2 = 0.031**

–; +weak; ++moderate; +++strong staining.

*PR*, Positive rate.

ref. reference.

*P1*:SG as ref. AG vs. SG.

*P2*:SG as ref. GC vs. SG.

**Table 3:** XPG expression in SG, AG of adjacent tissue and GC.

Group	Group1		Group2		Group3		Group4	
	Adjacent	GC	coupled-SG	coupled-GC	Adjacent-SG	AdjacentAG	coupled-AG	coupled-GC
Cases	131	176	88	88	88	41	41	41
(–)	74(56.5)	26(14.8)	69(78.4)	14(15.9)	69(78.4)	3(7.3)	3(7.3)	4(9.8)
(+)	27(20.6)	69(39.2)	15(17.0)	35(39.8)	15(17.0)	12(29.3)	12(29.3)	19(46.3)
(++)	18(13.7)	53(30.1)	2(2.3)	26(29.5)	2(2.3)	16(39.0)	16(39.0)	8(19.5)
(+++)	12(9.2)	28(15.9)	2(2.3)	13(14.8)	2(2.3)	10(24.4)	10(24.4)	10(24.4)
PR (%)	43.5	85.2	21.6	84.1	21.6	92.7	92.7	90.2
P	ref.	**<0.001**	ref.	**<0.001**	ref.	**<0.001**	ref.	0.244

–; +weak; ++moderate; +++strong staining.

*PR*, Positive rate.

ref. reference.

### Associations between XPG staining and clinicopathological characteristics

We analysed the associations between XPG expression and various clinicopathological parameters using Mann-Whitney U-tests (Table 4). XPG expression in intestinal-type GC (98.4%) was significantly higher than in diffuse-type GC. XPG expression levels were also significantly correlated with drinking (*P* = 0.031) (75.0%), depth of tumour invasion (pT stage, *P* = 0.012), macroscopic type (*P* = 0.032) (Table 4), *H. pylori* infection status (*P* = 0.039) and family history of cancer (*P* = 0.019) (Table S1). High XPG expression was observed in patients who drank, T4 cases, intestinal-type GC, *H. pylori* infection-positive, and family history-positive groups. However, there was no significant correlation between XPG expression and Borrmann classification, TNM stage, lymph node metastasis, growth pattern or lymphatic invasion (Table 4).

**Table 4:** Association between XPG expression and clinicopathological parameters in GC.

	Cases(n)	XPG expression					*p*
Variability		(–)	(+)	(++)	(+++)	PR (%)	
Macroscopic Type							**0.032**
Early stage	29	8	12	7	2	72.4	
Borrmann I–IV	138	18	54	43	23	87	
Borrmann classification							
Borrmann I–II	23	4	10	6	3	82.6	0.296
Borrmann III–IV	115	14	44	37	20	87.8	
Lauren’s classification							**<0.001**
intestinal-type	63	1	20	22	20	98.4	
Diffuse-type	96	24	41	25	6	75.0	
TNM stage							0.111
I–II	80	15	32	26	7	81.3	
III	87	11	34	24	18	87.4	
Lymph node metastasis							0.290
Positive	105	13	43	31	18	87.6	
Negative	61	12	23	19	7	80.3	
Depth of invasion							**0.012**
T1	27	7	12	7	1	74.1	
T2	27	5	10	10	2	81.5	
T3	23	4	13	4	2	82.6	
T4	9	10	31	29	20	88.9	
Growth pattern							0.191
Expanding	23	2	10	7	4	91.3	
Intermediate	78	10	30	23	15	87.2	
Infiltrative	66	14	26	20	6	78.8	
lymphatic invasion							0.480
Positive	33	4	18	6	5	87.9	
Negative	134	22	48	44	20	83.6	
family history							
Positive	32	2	10	13	7	93.8	
Negative	135	24	56	37	18	82.2	**0.019**

PR, Positive rate.

### Relationship between XPG expression and overall survival in patients with GC

We investigated the relationship between XPG expression and survival in patients with GC. According to univariate survival analysis, the expression level of XPG was not an independent prognostic factor (*P* = 0.491), while macroscopic type (*P* = 0.002), TNM stage (*P*<0.001), lymph node metastasis (*P*<0.001) and depth of invasion (*P*<0.001) were all significant prognostic factors (Table 1). Because TNM stage already included information on lymph node metastasis and depth of invasion, we performed multivariate analysis using Cox’s proportional hazards model adjusted by sex, age, TNM stage and macroscopic type. Interestingly, the results indicated that XPG expression level was an independent prognostic factor (*P* = 0.020, HR = 0.394, 95%CI 0.179–0.866). Patient with positive expression had a longer survival. We stratified the patients according to age and sex to elucidate more detailed relation between XPG and GC prognosis. Stratification analysis suggested patients aged younger than 60 years, who had positive XPG expression was significantly more favorable in terms of survival than that of patients with negative XPG expression (Figure S1); XPG expression was a protective factor no matter univariate survival analysis or Cox’s proportional hazards model (P = 0.021, HR = 0.373, 95%CI 0.154–0.901; P = 0.021, HR = 0.361, 95%CI 0.147–0.888 respectively), and male patients with XPG positive expression had significantly favorable overall survival (P = 0.021, HR = 0.373, 95%CI 0.154–0.901) (Table 5).

**Table 5:** Correlation between XPG expression and survival in GC.

	Cases	Cases ofEvents	MST	Univariate			Multivariate		
				P	HR	95%CI	P	HR	95%CI
XPG expression									
negative	26	8	29.070*		1(ref)			1(ref)	
positive	143	33	32.784*	0.296	0.666	0.307–1.442	**0.020**	0.394	0.179–0.866
Stratification									
Age <60									
Negative	16	7	31.000		1(ref)			1(ref)	
Positive	81	17	32.366*	**0.021**	0.373	0.154–0.901	**0.027**	0.361	0.147–0.888
Age ≥60									
Negative	10	1	34.714*		1(ref)			1(ref)	
Positive	62	16	32.306*	0.300	2.772	0.368–20.911	0.960	1.060	0.108–10.372
Gender									
Male									
Negative	14	5	27.571*		1(ref)			1(ref)	
Positive	99	21	33.454*	0.135	0.484	0.182–1.285	**0.002**	0.209	0.077–0.571
Female									
Negative	12	3	30.562*		1(ref)			1(ref)	
Positive	44	12	30.979*	0.859	1.120	0.316–3.970	0.800	0.841	0.219–3.225

MST median survival time.

*mean survival time.

HR: hazard radio, CI: confidence interval.

## Discussion

In the current study, we detected XPG protein expression in tissues from patients with SG, AG and GC, and in adjacent non-tumour tissues, by immunohistochemical staining. Moreover, we investigated the relationships between XPG protein expression and clinicopathological parameters and survival in GC patients, to provide insights into its roles in the development, progression and prognosis of GC. To our best of our knowledge, this is first report of a relationship between XPG protein expression and the development, progression and prognosis of GC.

A variety of underlying mechanisms might influence the expression of XPG, including *ERCC5* gene mutation, regulation of transcription and translation, protein degradation and promoter methylation [15]. The physiological regulation of XPG expression requires external stimulation of DNA damage. For example, UVC-induced DNA damage may up-regulate XPG expression [16]. In normal individuals DNA damage is rare, and the DNA repair gene *ERCC5* is therefore expressed at low levels. However, various types of environmental carcinogens and endogenous metabolic products may cause DNA damage, thus enhancing the DNA-repair activity of cells and the activities of transcription and translation [17]. The current study explored the XPG protein expression profile in the SG→AG→GC disease sequence and found XPG expression in SG was relatively lower than GC and AG. The results indicated that XPG protein was induced and activated during the process of carcinogenesis, thereby repairing damaged DNA and maintaining the integrity of the genome. XPG was up-regulated in GC tissues, revealing a potential role for XPG protein as a biomarker to predict the risk of GC and its precancerous lesions. A few studies to date have reported on the relationships between XPG protein expression and other cancers, and the results differed from our findings. For instance, Cheng et al. observed low XPG expression in peripheral blood leukocytes in patients with lung, head and neck, and breast cancers [18], [19], [20], [21], [22], [23]. XPG was deficient or downregulated in carcinoma of the testis and breast cancer [9], [24]. The controversial conclusions from these different studies might result from the diverse biological characteristics of the tumors studied, or from differences in detecting methods and sample sizes. Further large-scale investigations of XPG expression in different cancers are needed to confirm its role.

We further investigated the relationships between XPG expression and clinicopathological parameters including TNM stage, depth of invasion, nodal metastasis, macroscopic type, lymph vessel invasion and growth pattern. The results suggested that XPG protein expression was associated with depth of invasion and macroscopic type; Invasion of cancer cells into the subserous adjacent tissue and more advanced macroscopic type were both key factors with great impacts on disease progression. Previous studies reported that overexpression of DNA repair gene was positively related to deeper invasion and a more developed classification of GC. Ganzinelli M et al. suggested that malignant transformation was associated with the upregulation of genes involved in DNA repair and maintaining genomic stability [25]. It was suggested that long-term hypoxia and inflammation in the tissue microenvironment may be responsible for inducing DNA damage [26]. Furthermore, it has been reported that XPG genes were significantly less expressed in stage III than in stage I ovarian carcinoma [25]. Liu et al. suggested that ERCC1 mRNA expression levels was correlated with age, with high ERCC1 expression being more common in younger patients [27]. The different outcomes of diverse investigations might result from differences in cancer types, ethnicities, sample sizes and environmental factors. Our results indicated that strong expression was frequently detected in T4 and advanced cancer. Considering XPG was less expressed in diffuse-type GC than in intestinal-type GC, poorly differentiated cancer cells may lack the ability to generate XPG which was responsible for tissue repair. Diffuse-type GC might therefore have a poorer prognosis. The above evidence indicates that XPG expression was positively associated with a number of clinicopathological parameters reflecting GC development, and might thus play important roles in the initiation and progression of GC and serve as a biomarker for GC development, predicting biological activities and degree of progression. In addition, XPG overexpression was also associated with family history, *H. pylori* infection and drinking. Alcohol consumption and *H. pylori* infection may induce oxidative damage, thus increasing expression of DNA-repair proteins such as XPG. XPG was more highly expressed in patients with a family history of cancer, suggesting that it might also be a genetic biomarker of cancer.

We further investigated the relationships between XPG expression and overall survival. There was a significant association between XPG protein expression and GC prognosis in multivariate analysis, especially in patients aged younger than 60 years. Positive expression levels of XPG protein could predict longer survival according to the present study. Similarly, high expression of DNA-repair family proteins, such as *ERCC1*, predicted longer overall survival compared with low ERCC1 expression [27]. High XPG expression was associated with longer survival in patients with ovarian cancer [28]. In terms of mRNA levels, high XPG mRNA levels were an independent prognostic factor predicting longer survival in patients with non-small-cell lung cancer and sarcoma [29], [30]. In contrast, XPG has recently been reported to have prognostic value in ovarian cancer; low XPG expression predicted longer survival [31], in accordance with our current findings. Low expression levels of some genes for DNA-repair family proteins, such as *ERCC1*, have been reported to predict longer relapse-free survival and overall survival in GC. High XPF expression was related to early progression; patients with high XPF expression had shorter progression-free survival than patients with low XPF expression [32]. Liu et al. demonstrated that patients with low ERCC1 mRNA expression levels had longer relapse-free and overall survival times than patients with high ERCC1 levels. Different types of cancers have distinct mechanisms of carcinogenesis and their control thus differs between different populations. The prognostic role of XPG is therefore also likely to vary among different types of cancers. In addition, XPG expression might be influenced by various factors, and further large-scale multicentre investigations with a long follow-up are required to clarify the relevance of XPG in cancer prognosis. Nevertheless, XPG expression appears to have potential prognostic value in GC, especially in patients aged younger than 60 years, though further studies are needed to clarify the underlying mechanisms.

In conclusion, we demonstrated for the first time that XPG protein expression was significantly higher in GC than non-tumour tissues, and significantly higher in AG and GC than in SG in the disease sequence SG→AG→GC. The level of XPG expression was also significantly associated with depth of tumour invasion, macroscopic type, Lauren’s classification, smoking, *H. pylori* infection and family history of cancer. Multivariate survival analysis indicated that patients with XPG positive expression had significant longer overall survival, especially in patients younger than 60 years. Our results suggest that XPG protein expression is related to the development, progression and prognosis of GC, and may therefore serve as a potential biomarker for the diagnosis and prognosis of this disease.

## Supporting Information

Figure S1 A, correlation of XPG expression with survival curves of patients with gastric cancer by univariate survival analysis; B, correlation of XPG expression with survival curves of patients younger than 60 years in gastric cancer by univariate survival analysis; C, correlation of XPG expression with survival curves of patients olderer than 60 years in gastric cancer by univariate survival analysis.
(TIF)

Table S1 **Baseline characteristics of the study population and expression of XPG.**
(DOC)
