# Supplementary figures and images for: Expression of XPG Protein in the Development, Progression and Prognosis of Gastric Cancer
Source: PLoS One. 2014 Sep 30;9(9):e108704. doi: 10.1371/journal.pone.0108704 (PMC4182552; doi:10.1371/journal.pone.0108704)

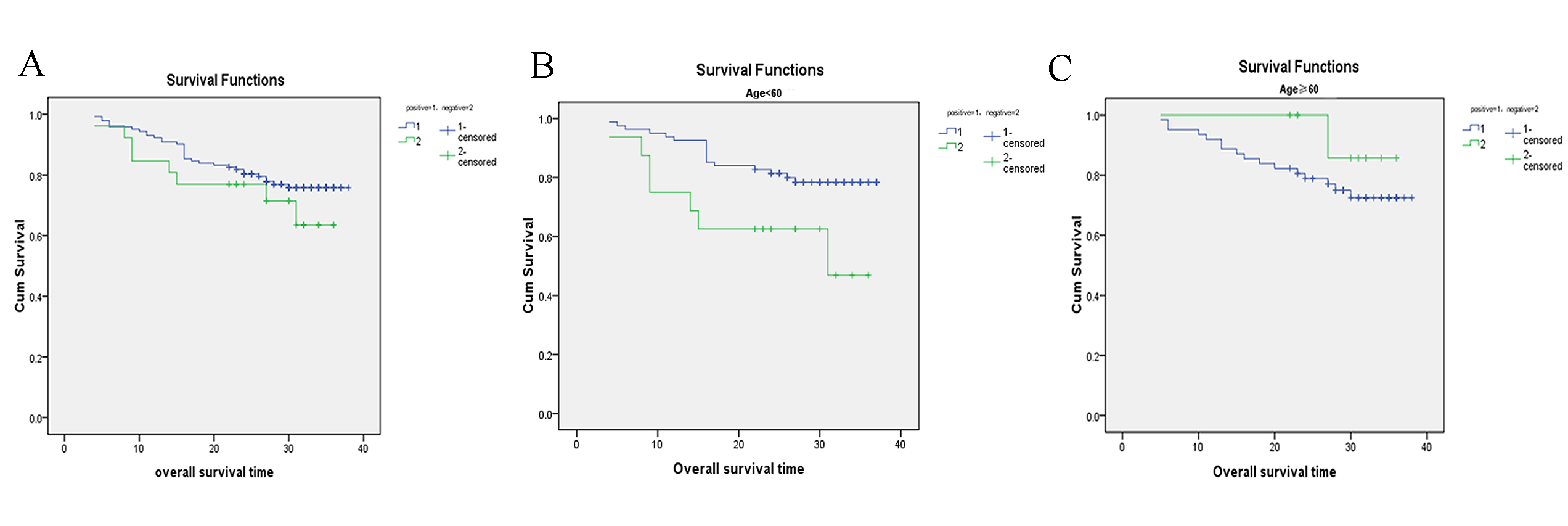

Supplement: Figure S1 — A, correlation of XPG expression with survival curves of patients with gastric cancer by univariate survival analysis; B, correlation of XPG expression with survival curves of patients younger than 60 years in gastric cancer by univariate survival analysis; C, correlation of XPG expression with survival curves of patients olderer than 60 years in gastric cancer by univariate survival analysis. (TIF) [file pone.0108704.s001.tif]
