# Supplementary material for: Expression of XPG Protein in the Development, Progression and Prognosis of Gastric Cancer
Source: PLoS One. 2014 Sep 30;9(9):e108704. doi: 10.1371/journal.pone.0108704 (PMC4182552; doi:10.1371/journal.pone.0108704)
Supplement: Table S1 — Baseline characteristics of the study population and expression of XPG. (DOC) [file pone.0108704.s002.doc]

| **Baseline characteristic of the study population and expression of XPG** | | | | | | | |
| --- | --- | --- | --- | --- | --- | --- | --- |
| Variability | Cases(n) | XPG expression | | | |  | *p* |
|  |  | (-) | (+) | (++) | (+++) | PR(%) |  |
| Gender |  |  |  |  |  |  | 0.368 |
| Male | 177 | 20 | 74 | 46 | 37 | 88.7 |  |
| Female | 101 | 16 | 39 | 31 | 15 | 84.2 |  |
| Age |  |  |  |  |  |  | 0.580 |
| <60 | 158 | 23 | 57 | 47 | 31 | 85.4 |  |
| ≥60 | 120 | 13 | 56 | 30 | 21 | 89.2 |  |
| Smoking |  |  |  |  |  |  | 0.305 |
| Yes | 110 | 12 | 45 | 28 | 25 | 89.1 |  |
| No | 168 | 24 | 68 | 49 | 27 | 85.7 |  |
| Drinking |  |  |  |  |  |  | **0.031** |
| Yes | 73 | 4 | 33 | 13 | 23 | 93.8 |  |
| No | 205 | 32 | 80 | 64 | 29 | 84.4 |  |
| HP infection status | |  |  |  |  |  | **0.039** |
| positive | 78 | 6 | 28 | 22 | 22 | 92.3 |  |
| negetive | 61 | 7 | 31 | 12 | 11 | 88.5 |  |
